# Evasion of Immune Surveillance in Low Oxygen Environments Enhances Candida albicans Virulence

**DOI:** 10.1128/mBio.02120-18

**Published:** 2018-11-06

**Authors:** José Pedro Lopes, Marios Stylianou, Emelie Backman, Sandra Holmberg, Jana Jass, Rolf Claesson, Constantin F. Urban

**Affiliations:** a Department of Clinical Microbiology, Umeå University, Umeå, Sweden; b Umeå Centre for Microbial Research, Umeå, Sweden; c Laboratory for Molecular Infection Medicine Sweden (MIMS), Umeå, Sweden; d Life Science Centre-Biology, School of Science and Technology, Örebro University, Örebro, Sweden; e Section Oral Microbiology, Department of Odontology, Umeå University, Umeå, Sweden; Duke University Medical Center

**Keywords:** *Candida albicans*, neutrophil, PMN, anoxia, hypoxia, immune evasion, fungal masking, fungal cell wall, abscesses, beta-glucan, mycology

## Abstract

Microbial colonizers of humans have evolved to adapt to environmental cues and to sense nutrient availability. Oxygen is a constantly changing environmental parameter in different host tissues and in different types of infection. We describe how Candida albicans, an opportunistic fungal pathogen, can modulate the host response under hypoxia and anoxia. We found that high infiltration of polymorphonuclear leukocytes (PMNs) to the site of infection contributes to a low oxygen milieu in a murine subdermal abscess. A persistent hypoxic environment did not affect viability or metabolism of PMNs. Under oxygen deprivation, however, infection with C. albicans disturbed specific PMN responses. PMNs were not able to efficiently phagocytose, produce ROS, or release extracellular DNA traps. Failure to launch an adequate response was caused by C. albicans cell wall masking of β-glucan upon exposure to low oxygen levels which hindered PAMP sensing by Dectin-1 on the surfaces of PMNs. This in turn contributed to immune evasion and enhanced fungal survival. The cell wall masking effect is prolonged by the accumulation of lactate produced by PMNs under low oxygen conditions. Finally, adaptation to oxygen deprivation increased virulence of C. albicans which we demonstrated using a Caenorhabditis elegans infection model.

## Abstract (precis)

Successful human colonizers have evolved mechanisms to bypass immune surveillance. Infiltration of PMNs to the site of infection led to the generation of a low oxygen niche. Exposure to low oxygen levels induced fungal cell wall masking, which in turn hindered pathogen sensing and antifungal responses by PMNs. The cell wall masking effect was prolonged by increasing lactate amounts produced by neutrophil metabolism under oxygen deprivation. In an invertebrate infection model, C. albicans was able to kill infected C. elegans nematodes within 2 days under low oxygen conditions, whereas the majority of uninfected controls and infected worms under normoxic conditions survived. These results suggest that C. albicans benefited from low oxygen niches to increase virulence. The interplay of C. albicans with innate immune cells under these conditions contributed to the overall outcome of infection. Adaption to low oxygen levels was in addition beneficial for C. albicans by reducing susceptibility to selected antifungal drugs. Hence, immunomodulation of host cells under low oxygen conditions could provide a valuable approach to improve current antifungal therapies.

## INTRODUCTION

A small proportion of known microorganisms can infect humans, and of those, only a minority is able to cause systemic disease. To do so, microbes need to adapt to complex host niches which demand for metabolic modifications and strategies to evade immune surveillance. Some of these adaptations involve adjusting to nutrient quality or scarcity and adjusting to stresses, such as different oxygen levels (1, 2). The oxygen levels inside the body vary from oxygen-rich (normoxic, 20% O_2_) to oxygen-depleted (anoxic, <0.2% O_2_) niches (3). Oxygenated directly by the ambient air, the lung alveolar epithelium has oxygen levels of 15%, whereas in the case of infected tissues, processes including accumulation of mucus, formation of abscesses, and generation of microbial biofilms create oxygen-poor niches with oxygen levels ranging below 0.6% (4–6).

One of these omnipresent microbes that are able to colonize such niches is Candida albicans. Even though C. albicans is a widely distributed commensal microorganism of humans, it is also the most common opportunistic fungal pathogen. The major reservoirs of C. albicans are the gastrointestinal, oral, and genital mucosa. As these different niches vary considerably in environmental conditions and microbiotic composition (7, 8), C. albicans has developed different metabolic strategies and growth forms, such as biofilm formation, which allow establishing perseverance and antifungal resistance (9–11). Interestingly, some genes important for biofilm formation are also essential for hypoxia adaptation in *Candida* species (12, 13).

Upon infection, low oxygen conditions arise under which immune cells, similarly to microbes, must maintain functionality to restore homeostasis (14, 15). As the first line of defense, a high number of polymorphonuclear leukocytes (PMNs) infiltrate the tissue. PMN extravasation coupled with the proliferation of other immune cells and the activation of oxygen-consuming enzymes fuel the creation of environments with decreasing oxygen levels (16).

How different oxygen levels influence the infectivity of microbes and how these levels modulate immune responses remain poorly understood. Here we show that infiltration of PMNs creates a favorable environment for C. albicans. Reduced oxygen levels are exploited by C. albicans to enhance pathogenesis via masking of β-glucan in the fungal cell wall. C. albicans can subsequently obscure PMN recognition and hence impede clearance, leading to establishment of successful infection. This was confirmed by infection experiments in mice and nematodes demonstrating that low oxygen levels enhance C. albicans infectivity and virulence. Adaption to low oxygen environments might contribute to the ability of C. albicans to colonize various niches, and studying these adaptation mechanisms provides insight into infection strategies of *Candida* species.

## RESULTS

### Candida albicans infection induced hypoxia.

Niches commensally colonized by C. albicans vary considerably in oxygen levels (17, 18). The yeast major reservoir is in the gastrointestinal tract, as well as the oral and genital mucosa (7). We first determined the levels of hypoxia in uninfected mice to identify tissues with naturally low oxygen levels. To quantify hypoxia generated in mice, we took advantage of the dye HypoxiSense which produces a fluorescent signal under hypoxic conditions. The reagent was successfully applied to study hypoxic processes in tumors and recently also to evaluate the onset of hypoxia during experimental pulmonary *Aspergillus fumigatus* infection (19, 20).

**FIG 1:**
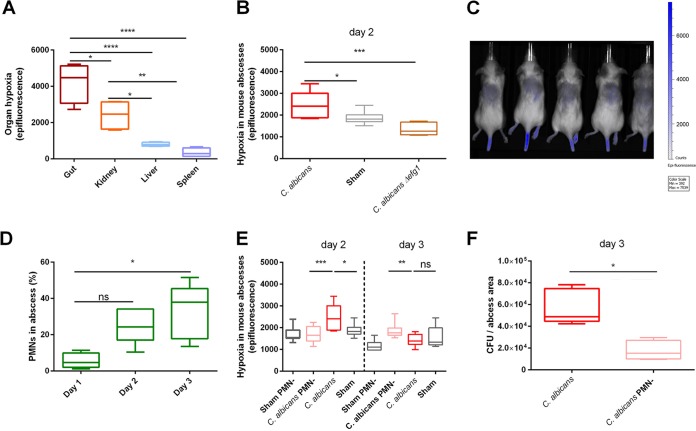
Hypoxia was induced in subdermal abscesses. (A) Quantification of basal hypoxia in the organs of uninfected mice. The gut and kidney showed the highest levels of hypoxia. Mice were injected i.p. with HypoxiSense dye, and after 24 h, hypoxia was monitored by fluorescence quantification using an IVIS camera system. Data are from four mice. (B) C. albicans subdermal infection induced hypoxia in an abscess at 48 h p.i. C. albicans mutants lacking the hypoxic regulator Efg1p were unable to induce hypoxia. Hypoxia was detected using HypoxiSense as described above, and mice were monitored by IVIS. *n* = 8 mice per group. (C) Representative image of C. albicans-infected mice assessed by IVIS. The region of interest (ROI) was drawn around the dorsal area of the mice where C. albicans was inoculated. Values for average counts of epifluorescence were used in the subsequent analyses. Background noise was subtracted from all animals by drawing an additional ROI. (D) PMNs infiltrate the abscess during subdermal infection with C. albicans. Infiltration from dissected abscesses of 5 mice per group was analyzed by flow cytometry after staining with anti-Ly6G-FITC. The percentage of PMNs is shown normalized to Ly6G-positive cells. For each sample, a total of 20,000 cells per abscess were analyzed. (E) Hypoxia was evaluated by IVIS at 48 h and 72 h after subdermal infection. At 48 h p.i., C. albicans-induced abscesses were hypoxic, whereas depletion of PMNs abrogated hypoxia induction. While abscesses from untreated mice lost hypoxia after 72 h, abscesses from depleted mice (PMN-) became hypoxic at 72 h p.i. (F) Colony-forming units (CFU) from C. albicans abscesses of 5 mice were approximately twofold above CFU from abscesses harvested from 4 PMN-depleted (PMN-) mice. Fungal burden is presented as CFU per mm^2^ of abscess area. Abscesses were collected at day 3 p.i. ns, not significant; *, *P* < 0.05; **, *P* < 0.01; ***, *P* < 0.001; ****, *P* < 0.0001.

Interestingly, commensal niches and some organs typically colonized by C. albicans during disseminated infection, such as the kidney, were more hypoxic than other internal organs (Fig. 1A). We reasoned that the ability to colonize and persist in low oxygen niches could be important to establish candidiasis. To test this, we used a subdermal infection in adult albino C57BL/6J mice, which resulted in abscess formation and conducted a similar hypoxia measurement. We chose a local abscess model over systemic infection, because of the local and acute onset of the infection. This model, therefore, allows us to determine oxygen levels in a confined space where C. albicans interacts with epithelial cells and infiltrating PMNs without secondary signals from systemic cytokine storms which arise in cases of disseminated infection. Hypoxia was established at 48 h postinfection (p.i.) (Fig. 1B and C) and subsided the following day. We tested the ability of C. albicans in a liquid culture to consume oxygen. C. albicans could consume oxygen only in a limited manner (see Fig. S2A in the supplemental material). Additionally, the C. albicans Δ*efg1* strain was unable to induce hypoxia in the subdermal abscess model (Fig. 1B). *EFG1* is important for biofilm and hypoxia regulation (12, 21, 22). We conclude that generation of hypoxia was related to host immunity.

Mice resolved the subdermal C. albicans infection after 5 to 7 days (Fig. S2B and C). Clearance was supported by the influx of immune cells. We quantified PMN infiltration to abscesses (Fig. 1D and Fig. S2D). As expected, PMNs were abundant in abscesses. The presence of PMNs increased from 5% to 40% of cellular content within 3 days of infection. Quantification of PMNs using histological sections of mouse abscesses showed similar percentages of PMNs after 5 days of infection (Fig. S3A). To determine the role of PMNs in the generation of hypoxia, we depleted PMNs prior to infection using an anti-mouse Ly6G antibody, an immunoglobulin commonly applied to deplete this cell type (23, 24). Depletion of PMNs blocked hypoxia induction at 48 h p.i. (Fig. 1E). Recovering PMNs in depleted mice restored hypoxia (Fig. 1E and Fig. S2E). Therefore, we concluded that high PMN influx to the site of infection was the main source of hypoxia. Interestingly, abscesses from PMN-depleted mice had areas similar to those of nondepleted mice (Fig. S2F and G). However, the fungal burden of abscesses from PMN-depleted mice was lower than in nondepleted mice. This is most likely due to differences in the prevalent growth morphotype adopted by C. albicans (Fig. 1F). Nevertheless, even in the presence of large numbers of PMNs, considerable viable counts of C. albicans persevered over a period of several days.

We conclude that outside commensal niches recruitment of PMNs, induced by C. albicans, generated hypoxia.

### Anoxia reduced C. albicans phagocytosis and killing by PMNs.

**FIG 2:**
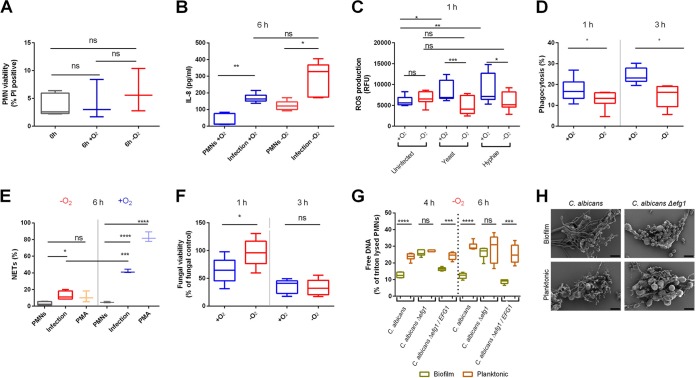
C. albicans escaped from PMN attack under anoxia. (A) Anoxia did not affect PMN viability. Uninfected PMNs were double stained with labeled anti-CD66b antibody and propidium iodide (PI). Cell viability was determined by measuring the number of double-positive cells after 0 h and 6 h using a flow cytometer. Data from *n* = 3 biological replica in triplicate. (B) IL-8 release was not different in anoxic or normoxic environments. IL-8 release from PMNs was quantified under normoxic and anoxic conditions using a Luminex ELISA. Supernatants of C. albicans-infected and uninfected samples at 6 h were assessed. Data from *n* = 3 biological replica in triplicate. (C) ROS production was undetectable in PMNs under anoxia. ROS production was induced with different C. albicans morphotypes and assessed using a fluorescence assay at 1 h and 3 h. Data were plotted as relative fluorescence units (RFU). (D) PMN phagocytosis of C. albicans was diminished under anoxic conditions. After 1 h and 3 h of incubation with GFP-expressing C. albicans, PMNs were stained with labeled anti-CD66b antibody. Double-positive cells were counted as phagocytosing cells using flow cytometry. Results are plotted as the percentage of phagocytosing cells normalized to the total number of PMNs analyzed. Data from *n* ≥ 5 biological replica in triplicate. (E) NET induction of PMNs stimulated by C. albicans was reduced in anoxic environments at 6 h. PMA, a ROS-dependent NET inducer, served as control. NET quantification was performed using ImageJ software, and objects with areas above 100 μm^2^ were counted as NET. Plotted are the percentages of NET events normalized to the total number of objects analyzed, *n* ≥ 3 biological replica and at least 10 objects per replica. (F) C. albicans resisted PMNs temporarily under anoxic conditions. Fungal survival in the presence of PMNs was determined by ATP quantification after 1 h and 3 h. Prior to ATP quantification, PMNs were lysed using detergent. Survival was plotted as percentage of the respective fungal control incubated without PMNs at the respective time point and oxygen condition. Data from *n* = 3 biological replica in triplicate. (G) C. albicans biofilm formed under anoxic conditions impaired NETosis. NET induction was assessed for 4 h or 6 h after exposure to biofilms or planktonic cultures of different C. albicans strains (wt*, Δefg1, Δefg1*/*EFG1*). NET inhibition was estimated by the detection of free DNA using membrane impermeable DNA dye Sytox Green. NET formation was plotted as a percentage of free DNA from Triton-lysed PMNs. Data from one representative experiment, in quintuplicate. (H) Analysis of NET formation under anoxic conditions using SEM. PMNs released NETs when exposed to planktonic C. albicans but not to biofilms of C. albicans (left panels). PMN response against C. albicans Δ*efg1* strain was unchanged in response to planktonic cells or biofilms (right panels). NETs were visualized by SEM at a magnification of 2,500× after 6 h of infection. Size bars represent 10 μm. ns, not significant; *, *P* < 0.05; **, *P* < 0.01; ***, *P* < 0.001; ****, *P* < 0.0001.

PMNs perform four main activities when encountering pathogens: respiratory burst, phagocytosis, degranulation, and the release of extracellular traps (neutrophil extracellular traps [NETs]) (25). PMNs are dependent on the presence of oxygen to mount an efficient respiratory burst and to initiate antimicrobial activities. Therefore, we tested the effect of anoxia on the functionality of PMNs. First, we investigated how anoxia could affect PMN metabolism and function to exclude hypoxia as a cofounder in our work. We isolated a highly viable and pure PMN population (Fig. S3B) and tested the PMNs for viability, metabolic activity, and functionality under oxygen deprivation. After several hours under anoxic conditions, PMNs showed no notable differences in viability, metabolism, or phagocytosis of particles compared to PMNs under normoxic conditions (Fig. 2A and Fig. S3B to E). We conclude that hypoxia did not affect PMN functionality in a general fashion. By quantifying lactate secretion, we also confirmed that the glycolytic metabolism of PMNs rendered them well-suited for anoxia (Fig. S3E).

Next, we assessed more specific PMN responses toward C. albicans. Upon C. albicans infection, secretion of proinflammatory chemokine IL-8 was not reduced under anoxic compared to normoxic conditions, whereas basal secretion in the absence of stimulus was rather elevated in anoxia (Fig. 2B). Expectedly, ROS production was abolished in anoxia (Fig. 2C and Fig. S3F).

We further investigated the ability of PMNs to phagocytose C. albicans. We used a GFP reporter strain of C. albicans (26), stained PMNs with CD66b antibody, and quantified double positive cells by flow cytometry (Fig. 2D). The results indicated a decreased ability of PMNs to phagocytose C. albicans under anoxic conditions at both time points tested. In good agreement, PMNs showed a reduced capacity to kill C. albicans cells at early time points of *in vitro* infection (Fig. 2F and Fig. S3G). At later time points (3 h), killing of C. albicans under anoxic conditions reached levels similar to those under normoxic conditions (Fig. 2F and Fig. S3G). Notably, in subdermal abscesses, considerable viable counts of C. albicans remained for several days even in the presence of large numbers of PMNs (Fig. 1F).

Additionally, *Candida*-induced NET formation was reduced by 50% in anoxia and delayed as it occurred after 6 h p.i. (Fig. 2E and Fig. S4A to C). Moreover, the average NET area was diminished in anoxia compared to NETs under normoxic conditions (Fig. S4D). This hints to a decreased functionality toward C. albicans as NETs under oxygen deprivation might not be able to entrap microbes as efficiently as normoxic NETs. In addition, C. albicans biofilm formation was reduced in anoxia; however, biofilms generated in anoxia could still impair NET formation in a manner comparable to that of biofilms generated under normoxic conditions. This indicates that the immune-modulating capacity of C. albicans biofilms was maintained under anoxic conditions (Fig. 2G and H and Fig. S4E and F).

To investigate whether the reduction of PMN functionality was due to C. albicans or due to PMN biology, we decided to analyze abscesses collected from periodontal patients and stained for NETs. We identified NETs in response to multibacterial species present in the abscess (Fig. S4G). We also stimulated PMNs from healthy patients with Aggregatibacter actinomycetemcomitans leukotoxin and quantified NET formation under normoxic and anoxic conditions (27) (Fig. S4H). The ability of PMNs to form NETs in response to leukotoxin was likewise undisturbed in both normoxic and anoxic environments.

This indicates that interference with PMN functionality in response to C. albicans was predominantly induced by the yeast.

### Anoxia induced C. albicans cell wall masking.

**FIG 3:**
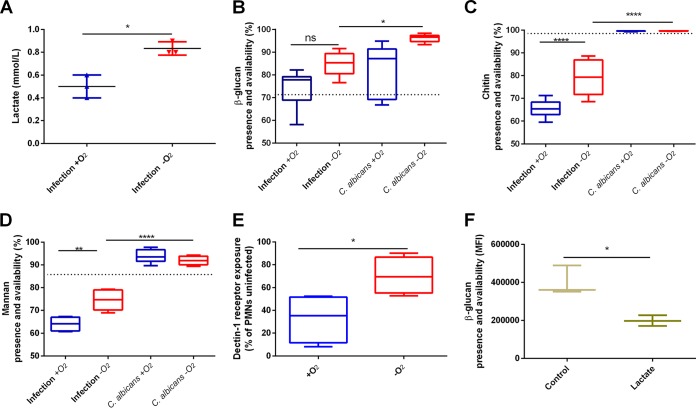
Anoxia induced fungal cell wall masking in C. albicans. (A) *In vitro* infection of PMNs with C. albicans induced PMNs to release higher levels of lactate under anoxic conditions than under normoxic conditions. Lactate in supernatants was analyzed at 6 h. Data from *n* = 3 biological replica in triplicate. (B) Presence and availability of β-glucan in the cell wall of C. albicans was assessed using staining with Fc-hDectin-1 conjugated to a fluorophore and analysis by flow cytometry after 2 h of incubation. Thimerosal-killed cells at time point 0 h served as a control (dashed line). Content of β-glucan was investigated in the presence (infection) or absence of PMNs. In the presence of PMNs, no significant change of β-glucan content could be observed under anoxic or normoxic conditions. Data from *n* = 3 biological replica in triplicate. (C) Similarly, the presence and availability of chitin in the cell wall of C. albicans was assessed in the presence and absence of PMNs using staining with CFW and analysis by flow cytometry after 2 h of incubation. Thimerosal-killed cells at time point 0 h served as a control (dashed line). In the presence of PMNs, chitin levels increased under anoxic conditions compared to normoxic conditions. Data from *n* = 3 biological replica in triplicate. (D) Similarly, the presence and availability of manna in the cell wall of C. albicans was assessed in the presence and absence of PMNs using staining with concanavalin A conjugated to a fluorophore and analysis by flow cytometry after 2 h of incubation. Thimerosal-killed cells at time point 0 h served as a control (dashed line). In the presence of PMNs, chitin levels increased under anoxic conditions compared to normoxic conditions. Data from *n* = 3 biological replica in triplicate. (E) Recognition of C. albicans by Dectin-1 on PMNs is decreased after 2 h under anoxic conditions. Dectin-1 receptor exposure on PMNs after anoxic and normoxic C. albicans in vitro infection was analyzed by flow cytometry. The graph shows percentage of receptor exposure on the surfaces of PMNs normalized to respective uninfected PMNs under anoxic and normoxic conditions. Receptor exposure was determined as the level of the mean of fluorescence intensity (MFI) after 2 h of incubation. Data from *n* = 4 biological replica in triplicate. (F) Prolonged exposure to PMN supernatants induced β-glucan masking. Flow cytometry analysis of the presence of β-glucan and availability in the cell wall of C. albicans after 7 h of incubation with supernatants from uninfected anoxic cultures of PMNs (20 h) was performed as described above for panel B). C. albicans cultured in RPMI served as a control. Data from *n* = 3 biological replica. ns, not significant; *, *P* < 0.05; **, *P* < 0.01; ***, *P* < 0.001; ****, *P* < 0.0001.

Alterations to the environment result in cell wall remodelling of C. albicans. Hypoxia and carbon source availability are known to influence the cell wall composition (28). Recently, the presence of lactate has been shown to induce cell wall masking on C. albicans cells which in turn compromised pathogen-associated molecular pattern (PAMP) exposure (29). We found that PMNs can be a source of lactate and that 40% increased amounts of lactate were available during *in vitro* infection under anoxic conditions (Fig. 3A). To elucidate whether attenuated phagocytosis was related to cell wall masking, we quantified three major fungal cell wall components β-glucan, chitin, and mannan. We infected PMNs with C. albicans *in vitro*. The presence and availability of β-glucan after 2 h under anoxic conditions in response to PMNs were not notably different from normoxic infection (Fig. 3B). However, levels of chitin and mannan were significantly higher during *in vitro* infection under anoxic than under normoxic conditions (Fig. 3C and D). To test whether altered chitin and mannan levels contributed to an indirect fungal masking of β-glucan, we stained for the Dectin-1 receptor, the C-type lectin receptor involved in β-glucan recognition (30). Upon binding of ligand β-glucan, Dectin-1 on PMNs is internalized for the activation of downstream signaling pathways and thus no longer exposed at the cell surface (31). Therefore, comparison of Dectin-1 surface exposure in uninfected PMNs to surface exposure after infection elucidates how well fungal cells are recognized by the host cell. Remarkably, 70% of Dectin-1 remained surface exposed in anoxic PMNs, indicating defective C. albicans recognition due to β-glucan masking (Fig. 3E). Since lactate signaling regulates fungal β-glucan exposure, we investigated whether the presence of lactate in supernatants harvested from PMNs incubated under anoxic conditions would be sufficient to cause β-glucan masking (29). Indeed, exposure to lactate-containing supernatants of anoxia-incubated PMNs caused a decrease in β-glucan presence and availability on C. albicans cell surfaces (Fig. 3F).

In low oxygen environments and in the presence of PMNs, C. albicans evaded phagocytes by cell wall masking. Prolonged exposure to low oxygen led to accumulation of lactate extending the masking effect.

### Low oxygen levels increased C. albicans virulence *in vivo*.

**FIG 4:**
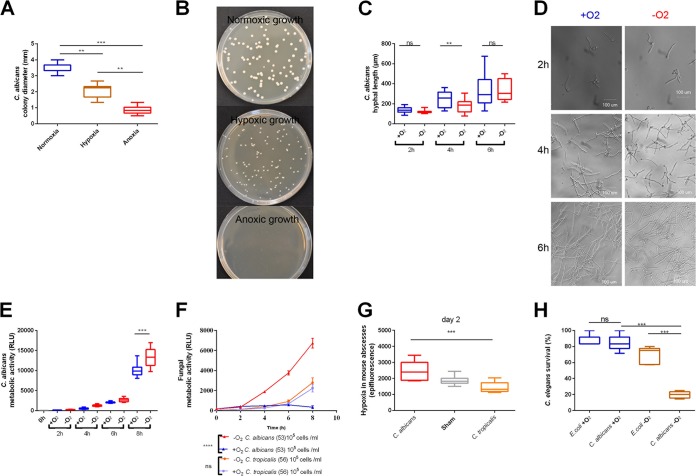
Low oxygen environments promoted C. albicans virulence. (A) Diameter of C. albicans colonies was evaluated under normoxia, hypoxia, and anoxia after 48 h. For 10 colonies from each plate, the diameters were measured and plotted as mean. Data from *n* = 3 biological replica with 10 colonies each. (B) Representative plates of C. albicans grown under normoxia, hypoxia, and anoxia for 48 h. Fungus was grown at an initial density of 1 × 10^2^ cells. (C) C. albicans hyphal elongation was transiently delayed in anoxic conditions compared to normoxic conditions. The hyphal length was determined using ImageJ after image acquisition using a Zeiss Axiovert 25 microscope. *n* ≥ 11 biological replica. (D) Microscopic assessment of C. albicans hyphal elongation in anoxic conditions compared to normoxic conditions. The fungus was grown at an initial density of 5 × 10^6^ cells/ml in 24-well plate, and images were taken at the indicated time points. Scale bars, 100 μm. (E) The metabolic activity of C. albicans is higher under anoxic conditions than normoxic conditions. Shown is a representative growth curve for C. albicans from 0 h to 8 h assessed by ATP quantification in 10 replicates. (F) Clinical isolates from C. albicans and C. tropicalis were grown under anoxic and normoxic conditions. Under anoxic conditions, C. albicans showed significantly higher metabolic activity than C. tropicalis did. A representative growth curve assessed by ATP quantification is depicted for both fungal species in 4 replicates. (G) C. albicans, but not C. tropicalis, induced hypoxic abscesses. Mouse (*n* = 8 animals per group) epifluorescent counts were measured 48 h p.i. by IVIS after injection of HypoxiSense. (H) Hypoxia increased virulence of C. albicans infection in C. elegans. Worms were cultured with E. coli or infected with C. albicans. Plates were incubated under normoxic or hypoxic conditions (1% oxygen level) for 48 h, and survival was determined as the percentage of live worms normalized to the total number of worms plated. Data represent *n* = 5 biological replica with 10 worms each. ns, not significant; **, *P* < 0.01; ***, *P* < 0.001.

Growth in low oxygen environments comes at the cost of slower fungal growth (Fig. 4A and B). Despite this fitness cost, the transition of yeast to hyphal growth, an essential trait for host invasion and virulence of C. albicans (32), was only transiently affected (Fig. 4C and D). We also analyzed the metabolic activity of C. albicans in anoxic and normoxic conditions by quantifying ATP levels, similarly as previously described (26, 33). Interestingly, adaptation to low oxygen environments was suggested by a high metabolic rate within the first 8 h of incubation for both type and clinical strains of C. albicans (Fig. 4E and Fig. S5A to E). We also observed this metabolic increment in clinical isolates of other *Candida* spp. isolated from candidemia patients (Fig. S5F to J) with the exception of Candida tropicalis isolates (Fig. 4F and Fig. S5K). C. tropicalis strains did not show increased metabolic activity under anoxic conditions.

To examine the relevance of high metabolism under hypoxic conditions during *in vivo* infection, we compared C. albicans with C. tropicalis using the subdermal abscess model. Despite the mice showing signs of infection, such as elevated temperature and weight loss (Fig. S6A and B), C. tropicalis abscesses were smaller than C. albicans abscesses, yet fungal burden in the abscess was similar to C. albicans infection, demonstrating that C. tropicalis was able to establish an infection. More importantly, in contrast to C. albicans infection, hypoxia generation was absent in C. tropicalis abscesses (Fig. 4G and Fig. S6C to E). This indicates that C. albicans-induced PMN recruitment caused hypoxia, whereas C. tropicalis infection did not.

We used an alternative infection model to test the contribution of hypoxia to C. albicans virulence. Several publications report that Caenorhabditis elegans can tolerate both anoxic and hypoxic environments (34–36). Different oxygen levels nevertheless could influence the physiology of the worms. Therefore, we have controlled our experiments with worms incubated with an avirulent Escherichia coli strain which C. elegans can use as a food source. Using the nematode model, we aimed to follow a course of infection under hypoxic conditions to demonstrate whether C. albicans virulence could increase. Preadult (L4 stage) worms were transferred to nematode growth medium (NGM) plates with C. albicans or E. coli OP50 and worms were monitored for survival. Remarkably, under hypoxic conditions (1% oxygen), C. albicans infection became more lethal for C. elegans than under normoxic conditions, killing more than 80% of the worms (Fig. 4H). Since a large proportion of the worms not infected with C. albicans survived under hypoxic conditions, we conclude that the plasticity to create and to adapt to low oxygen niches conferred an edge for C. albicans virulence.

As we observed a remarkable decrease of C. albicans recognition by PMNs and a significant increase of C. albicans virulence in a nematode model under hypoxic conditions, we aimed to investigate possible effects of hypoxia on exposure to current antifungal agents using a disc diffusion assay. Whereas antifungal activity of terbinafine was not affected by oxygen levels, the antifungal activity of fluconazole was considerably reduced under anoxic conditions (Fig. S6F and G). This indicates that adaption to hypoxia may additionally benefit C. albicans to escape from selected antifungal therapies.

## DISCUSSION

Low oxygen is a feature of infectious and physiological niches. These sites are zones of intense immune activity, and the impact of low oxygen on microbes and immune cells affects the outcome of the interplay. Dental pockets are such low oxygen zones where disease severity can be increased with the presence of C. albicans (37). Growing as a biofilm, C. albicans can support the growth of anaerobic bacteria in dental pockets (38). Immune responses in these niches are orchestrated by PMNs (39). Large numbers of PMNs are recruited to resolve the infection and to restore homeostasis. Microbes like A. actinomycetemcomitans have therefore developed strategies to disarm PMN responses, such as by the release of leukotoxin (40).

PMNs also constitute the first line of defense against fungal pathogens. In a subdermal abscess model of C. albicans infection in mice, granulocytes are the main immune cell type present (41). In agreement, we observed increasing infiltration of PMNs over time in a comparable infection model (Fig. 1D). While the previous study focused on encapsulation of C. albicans within the abscess, our main interest was on the impact of oxygen levels. Infiltration correlated well with the establishment of local hypoxia (Fig. 1B), and hypoxia could be abrogated by depletion of PMNs (Fig. 1E). Interestingly, CFU counts from abscesses of PMN-depleted mice were lower than counts from abscesses of nondepleted mice (Fig. 1F). The reduced CFU counts might at least partially stem from a more pronounced hyphal morphology of C. albicans in the abscess areas of PMN-depleted mice. Nevertheless, C. albicans remained in the abscess with considerable viable counts for several days in the presence of large numbers of PMNs. We suggest that induction of hypoxia in the abscess promoted C. albicans perseverance.

We examined how low oxygen levels modulated antifungal responses by PMNs *in vitro* mounted against different C. albicans morphotypes and against biofilms. In the absence of C. albicans, PMN viability and metabolism were unaffected by anoxia (Fig. 2A and Fig. S3D), confirming the results of a previous report (42). In addition, it has been shown that hypoxia hampers bacterial killing by PMNs (43). Therefore, we decided to assess the capacity of PMNs to phagocytose particle beads coated with Staphylococcus aureus components. We found no difference in phagocytic uptake of these particles under normoxic and anoxic conditions (Fig. S3D). PMN function is not hampered in anoxia *per se*; therefore, the reduction of phagocytic uptake depends instead on the microbe encountered. In compliance with this notion, specific PMN functions launched against C. albicans were compromised under anoxic conditions, such as ROS triggering, phagocytosis, and NET formation (Fig. 2). The capacity of C. albicans to form biofilms was somewhat reduced under anoxic conditions; however, as previously shown for biofilms generated under normoxic conditions (44), anoxically formed biofilms could inhibit PMN functions, such as the release of NETs. Of note, performing infections in anoxic environments allowed us to confirm ROS-independent mechanisms of NET induction (45).

**FIG 5:**
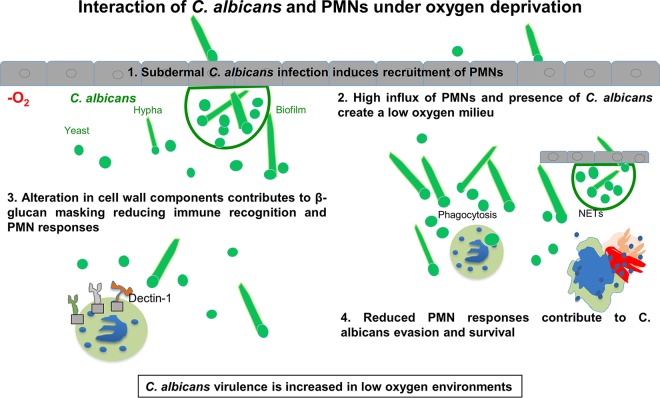
Summary model of interaction of C. albicans and PMNs under oxygen deprivation.

We observed that PMNs infected with C. albicans *in vitro* released more lactate under anoxic conditions than under normoxic conditions (Fig. 3A). Lactate has been previously shown to be important for β-glucan masking and subsequent immune evasion (29). Hence, we investigated whether reduced antifungal responses of PMNs toward C. albicans stem from cell wall masking. Indeed, in anoxia, β-glucan on the surface of C. albicans was masked due to alterations in mannan and chitin layers (Fig. 3B to D) as confirmed by reduced receptor internalization on PMNs (Fig. 3E). Dectin-1 on the surfaces of PMNs recognizes β-glucan. PMNs under anoxic conditions internalized far less Dectin-1 than the normoxic counterparts did, indicating that β-glucan exposure was reduced in C. albicans incubated under anoxic conditions (Fig. 3E). In addition, exposure to PMN supernatants containing lactate prolonged β-glucan masking on C. albicans cell walls (Fig. 3F). Therefore, it seems reasonable that low oxygen levels promote immune evasion of C. albicans (Fig. 5).

Interestingly, establishing hypoxia during initial colonization or infection is a strategy used by other microbes with a successful outcome (14, 46, 47). In good agreement with this notion, fungal adaptation to low oxygen seems to be linked to fungal pathogenesis, since null mutants of hypoxia regulators, such as *EFG1*, display attenuated virulence (11, 48, 49). Nevertheless, adaptation to low oxygen results in a fitness cost (Fig. 4A and B). Remarkably however, in the first hours of anoxic growth, C. albicans showed metabolic activity equal to or higher than that of normoxic strains (Fig. S5A to E).

C. tropicalis lacks homologues for low oxygen regulator *EFG1* (50) and failed to induce hypoxia in the subdermal abscess model (Fig. 4G). Although abscesses from C. tropicalis infection were smaller than abscesses from C. albicans infection, C. tropicalis was able to accumulate comparable fungal burden (Fig. S6C and D). In line with this, recent literature described C. tropicalis as a robust species with virulence attributes similar to those of C. albicans (51–53). A difference between the two species we note is the induction of hypoxia in the abscesses, allowing C. albicans to reduce susceptibility to PMN attack. In compliance, systemic C. tropicalis infections affect predominantly neutropenic patients, whereas systemic C. albicans infections are not clearly associated with neutropenia (54). This supports our findings that C. albicans adaption to low oxygen environments promoted evasion from PMN attack.

To further elucidate whether adaptation to low oxygen environments by *Candida* species might correlate with virulence, we took advantage of a nematode infection model. C. elegans is an established infection model for *Candida* spp. and has been shown to induce specific antifungal responses at low oxygen levels (34, 55). Here, we show that upon infection with C. albicans, worms were rapidly killed in hypoxia, whereas the majority survived under normoxic conditions (Fig. 4H). In addition, our data suggest that low oxygen levels in infection niches alter the susceptibility of C. albicans to certain antifungal drugs (Fig. S6F and G). These findings have implications for risk assessments of mycosis patients. Nevertheless, besides hampering PMN responses, low oxygen levels might diminish other forms of innate immune responses (56).

In conclusion, adaptation to hypoxia and anoxia appears to be a good example of adaptive prediction in C. albicans (57). In order to thrive, C. albicans takes advantage of low oxygen niches created during infection. As a result of the alternation of oxygen availability, C. albicans increases survival mainly by avoiding PMN attack via β-glucan masking and by evading ROS-dependent immune responses. This knowledge could have therapeutic value by inspiring novel immunomodulatory strategies to boost activities of immune cells under infection-induced hypoxia.

## MATERIALS AND METHODS

### *In vitro* infection under anoxic conditions.

*In vitro* experiments were performed in an anoxia incubation chamber (∼0.02% oxygen). The chamber was maintained at 37°C and kept at a low level of oxygen by utilizing a gas mixture containing 10% H_2_, 5% CO_2_, and 85% N_2_. The medium used during anoxic experiments was introduced in the chamber the day before the experiment to allow the exchange of residual oxygen present in solution. All cells were introduced as pellets in the chamber, and appropriate concentration dilutions were performed inside the anoxic chamber. To ensure oxygen-free pellets, we measured oxygen levels in cell pellets and solutions using a miniaturized low-range oxygen sensor (58) (STOX; Unisense). All control experiments performed under atmospheric levels of oxygen in the standard laboratory were considered normoxic.

### Microbial strain culture conditions.

The microbial strains used in this study are listed in Fig. S1 in the supplemental material. In all cases, C. albicans was incubated in synthetic complete dropout medium (SC medium) plus 2% glucose for 20 h at 30°C. A fresh subculture of C. albicans was inoculated in SC medium at 30°C for 3 h. Finally, the numbers of cells were counted and adjusted according to each experimental protocol. For filamentous forms, C. albicans was subcultured for 1 h, subsequently washed, counted, adjusted to 1 × 10^7^ cells/ml, and grown at 37°C in RPMI for 2 h. For some control experiments, heat-killed C. albicans cells were generated by exposure to 65°C for 30 min.

10.1128/mBio.02120-18.1 FIG S1 List of strains used in this work. Download FIG S1, PDF file, 0.4 MB. Copyright © 2018 Lopes et al. 2018 Lopes et al. This content is distributed under the terms of the Creative Commons Attribution 4.0 International license.

10.1128/mBio.02120-18.2 FIG S2 C. albicans, subdermal infection, and PMNs. (A) Growing C. albicans consumed oxygen initially, but consumption ceased after 10 min. C. albicans (1 × 10^7^ cells/ml) oxygen consumption was measured for 40 min using the STOX oxygen sensor. Data are from one representative experiment. (B) Mouse weight was assessed during subdermal C. albicans infection (*n* = 10 mice per group). (C) Surface temperature of mice was determined during subdermal C. albicans infection (*n* = 10 mice per group). (D) Percentage of PMNs with engulfed C. albicans extracted from subdermal abscesses increased over time. Abscesses were collected from sacrificed mice (*n* = 5), and PMNs (anti-Ly6G) and C. albicans (CFW) were stained and analyzed by flow cytometry. Phagocytosis was presented as a percentage of double-positive cells normalized to the total number of PMNs. For each sample, a total of 20,000 cells per abscess were analyzed. (E) At 72 h p.i., PMN-depleted mice (*n* = 4) do not differ from nondepleted mice (*n* = 5) in the number of PMNs in circulation. Blood was collected by heart puncture. PMNs were stained (anti-Ly6G-PE) and analyzed by flow cytometry. (F) Abscess area was measured 72 h p.i. using a caliper. No notable differences between the analyzed conditions were observed. Data from *n* = 5 mice for C. albicans infection, *n* = 4 mice for C. albicans infection in PMN-depleted animals, and *n* = 3 for C. tropicalis infection. (G) Representative images of abscesses (arrow) with C. albicans wild-type and *Δefg1* strains in mice depleted for PMNs or not depleted for PMNs 72 h p.i. Download FIG S2, TIF file, 0.7 MB. Copyright © 2018 Lopes et al. 2018 Lopes et al. This content is distributed under the terms of the Creative Commons Attribution 4.0 International license.

10.1128/mBio.02120-18.3 FIG S3 Analysis of PMNs in subdermal and *in vitro* infection. (A) Representative image (left) and quantification (right) of PMNs using histologic sections of subdermal abscesses from C. albicans-infected mice. A PMN-specific stain (fluorescently labeled Ly6B.2 monoclonal antibody clone 7/4; shown in red) was used, and all nucleated cells were stained with DAPI (shown in blue). Presented is the percentage of fluorescent pixel area (PMNs) normalized to DAPI signal (nucleated cells). Data are from *n* = 4 abscesses. (B) PMNs isolated from human blood are pure (97%) and viable (95.5%). PMNs were stained with anti-CD66-APC and PI and analyzed by flow cytometry. Data from *n* = 3 biological replica. (C) Metabolic activity at 6 h of incubation is not affected when comparing uninfected PMNs under normoxic or anoxic conditions using ATP quantification. Data from *n* = 4 biological replica in triplicate. (D) Phagocytic activity of PMNs was unchanged under anoxia and normoxia. PMN phagocytosis under anoxia was quantified using pHrodo particle conjugates for 1 h and 2 h. Cytochalasin D was added to control samples to inhibit the phagocytic process. Data from *n* = 3 biological replica in quadruplicate. (E) PMN metabolism is mainly glycolytic. Lactate concentration in cell supernatants was quantified at 6-h and 20-h incubation of PMNs under anoxic or normoxic conditions. Data from *n* = 3 biological replica in triplicate. (F) Upon stimulation with C. albicans yeasts or hyphae, PMNs are unable to produce ROS under anoxic conditions. To quantify ROS, the fluorescent dye CMH(2)CFDA was used, and data were plotted as RFU for each condition. (G) C. albicans temporally resisted killing by PMNs under anoxic conditions. Fungal killing by PMNs was quantified by CFU relative to the value for fungal control at 1 h and 3 h. Survival was plotted as a percentage of the respective fungal control incubated without PMNs at the respective time point and oxygen condition. Data from *n* = 4 biological replica in triplicate. Download FIG S3, TIF file, 0.5 MB. Copyright © 2018 Lopes et al. 2018 Lopes et al. This content is distributed under the terms of the Creative Commons Attribution 4.0 International license.

10.1128/mBio.02120-18.4 FIG S4 PMN response against C. albicans was reduced in anoxia. (A and B) PMNs released NETs upon stimulation with C. albicans under anoxic conditions. PMNs were infected with C. albicans (MOI of 1) *in vitro* for 6 h (A) or left untreated (B). Shown are representative micrographs of indirect immunofluorescence from fixed and permeabilized samples with fluorescence staining (DAPI) for DNA (blue) and fluorescence immunostaining for neutrophil elastase (green), myeloperoxidase (white), as well as C. albicans (red). NETs (arrows) were identified by colocalization of extracellular laminar DNA (blue) with elastase (green) and myeloperoxidase (white) around C. albicans filaments (red). Scale bars represent 20 μm. (C) Quantification of NET induction in anoxia at 4 h. NETs were quantified by analysis of microscopic images using ImageJ software, and objects with areas above 100 μm^2^ were counted as NET. Plotted are the percentages of NET events normalized to the total amount of objects analyzed, *n* ≥ 3 biological replica and at least 10 objects per replica. (D) NETs formed in anoxia are significantly smaller than when triggered under normoxic conditions. The average NET area was determined using representative images from anoxic and normoxic samples. (E) Comparison of the biofilm density of C. albicans under anoxic and normoxic conditions. Pregrown biofilms were stained with crystal violet and absorbance was measured. Data from *n* = 3 biological replica in quadruplicate. (F) Shown are representative microscopic images of C. albicans biofilms from different starting inoculates formed under anoxic and normoxic conditions and stained with crystal violet. Scale bar represents 100 μm. (G) Analysis of human abscess by indirect immunofluorescence microscopy. The abscess was collected from a patient with periodontitis from tooth material (tooth 12). The abscess contained the following: Actinomyces israelii (17%), Parvimonas micra (34%), *Fusobacterium* spp. (4%), and *Campylobacter* spp. (45%) as determined by MALDI-TOF. To display *in vivo* NETosis, samples were stained for DNA (blue) and neutrophil elastase (green). Scale bar represents 20 μm. (H) A. actinomycetemcomitans leukotoxin induced NET-like structures in anoxic and normoxic infected PMNs at 3 h. NET quantification was performed using ImageJ, *n* ≥ 3 biological replica and at least 10 objects per replica. Download FIG S4, TIF file, 0.9 MB. Copyright © 2018 Lopes et al. 2018 Lopes et al. This content is distributed under the terms of the Creative Commons Attribution 4.0 International license.

10.1128/mBio.02120-18.5 FIG S5 Analysis of metabolic activity of growing *Candida* strains under anoxic and normoxic conditions. (A to E) Metabolic activity of C. albicans assessed by ATP quantification at different starting inoculates as follows: 1 × 10^6^ cells/ml (A), 5 × 10^5^ cells/ml (B), 1 × 10^5^ cells/ml (C), 5 × 10^4^ cells/ml (D), and 1 × 10^4^ cells/ml (E). Data from *n* = 3 biological replica in quadruplicate. (F to K) Metabolic activity of different *Candida* spp. assessed by ATP quantification at different starting inoculates. The different *Candida* spp. were C. krusei (F), C. glabrata (G), C. lusitaniae (H), C. dubliniensis (I), C. guilliermondii (J), and C. tropicalis (K). Data from one representative experiment in quintuplicate. Download FIG S5, TIF file, 0.7 MB. Copyright © 2018 Lopes et al. 2018 Lopes et al. This content is distributed under the terms of the Creative Commons Attribution 4.0 International license.

10.1128/mBio.02120-18.6 FIG S6 Comparison of C. albicans and C. tropicalis in subdermal infection and susceptibility to current antifungal drugs in anoxia. (A) Weight of mice (*n* = 10 per group) subdermally infected with C. albicans or C. tropicalis. (B) Surface temperature of mice (*n* = 10 per group) subdermally infected with C. albicans or C. tropicalis. (C) Abscess areas were larger from mice infected with C. albicans (*n* = 5) than with C. tropicalis (*n* = 3). Abscesses were measured at 72 h p.i. using a caliper. (D) C. albicans*-*infected mice (*n* = 5) had fungal burdens similar to those of C. tropicalis*-*infected mice (*n* = 3). Fungal burden was presented as CFU per mm^2^ of abscess area. (E) Representative images of abscesses (arrow) from subdermal mouse infections with C. albicans or C. tropicalis at 72 h p.i. (F) Disc diffusion assay of C. albicans under anoxic and normoxic conditions. Data from *n* = 3 biological replica. (G) Representative image of an agar plate from the disc diffusion assay. Download FIG S6, TIF file, 0.8 MB. Copyright © 2018 Lopes et al. 2018 Lopes et al. This content is distributed under the terms of the Creative Commons Attribution 4.0 International license.

The purified leukotoxin protein was isolated from the culture supernatants of A. actinomycetemcomitans HK 1651 as described elsewhere (27).

### PMN isolation.

Blood samples were drawn from healthy individuals upon fully informed consent according to the principles stated in the Declaration of Helsinki and according to the instructions of the local ethical committee. PMNs were isolated from venous blood as previously described (59).

Dead human cells were quantified after staining with PI (Biosciences) in a FACS Accuri C6 instrument (BD Biosciences) with postacquisition data analysis with Accuri C6 analysis.

### Subdermal C. albicans infection in mice.

To test the formation of a hypoxic milieu in an abscess, we infected 6- to 8-week-old albino female mice [B6(Cg)-Tyrc-2J/J; Jackson Laboratory-USA] with C. albicans or C. tropicalis. For abscess induction, animals were anesthetized with 2.5% isoflurane (Orion Pharma, Abbott Laboratories Ltd., Great Britain), shaved on the dorsal side, and infected with 5 × 10^7^ cells in 100 µl subdermally. Sham-treated mice were injected with 100 µl PBS. For PMN depletion, 100 µg *InVivo*Plus anti-mouse Ly6G antibody (clone 1A8; BioXcell) was injected intraperitoneally (i.p.) into each mouse 1 day prior to infection.

All animals were subsequently injected with 2 nmol (100 µl) HypoxiSense 680 (Perkin Elmer) and monitored for hypoxia using IVIS (*In Vivo* Imaging Systems; Caliper Life Sciences, Inc.). HypoxiSense is a fluorescent dye which detects surface expression of carboxic anhydrase 9. This protein is upregulated in host cells under hypoxic conditions and thus can be used as a marker of oxygen levels present in tissues. Prior to imaging, the mice were anesthetized using the XGI-8 gas anesthesia system (Caliper Life Sciences, Inc.). At 72 h p.i., thr mice were sacrificed, the abscesses were dissected, and hypoxia was assessed. Surface temperature and body weight of the mice were determined daily. Surface temperature was assessed using a noncontact infrared thermometer. In all fluorescent pictures, a region of interest (ROI) was drawn over the dorsum over the area of subdermal infection. Average epifluorescence counts were used to determine the hypoxic status of the mice. An additional control ROI was drawn in the background and subtracted from all values. Analyses were performed using Living Image software version 4.5 (Perkin Elmer).

For flow cytometry analysis, six infected animals per day were sacrificed and abscesses were collected. Cells were prepared for flow cytometry analyses by digesting tissue with 25 U DNase I–0.1% collagenase A (Sigma-Aldrich) for 1 h at 37°C. The resulting digest was then filtered through a 70-µm cell strainer (Falcon) to remove debris. Cells were blocked with an anti-CD16/CD32 cocktail (BD Biosciences), and PMNs were stained using anti-Ly6G labeled with FITC or CD11b labeled with PE (BD Biosciences) and C. albicans 0.05% CFW. A total of 20,000 events were acquired in the flow cytometer for each sample.

The animal experiments were conducted in accordance with animal ethical guidelines stated in permission A79-14 from the Swedish Board of Agriculture.

### Mouse abscess immunohistochemistry.

For histology purposes, subcutaneous abscesses were removed after 5 days of infection. Tissue samples were fixed in 2% formalin, dehydrated, embedded in paraffin, and sliced into 5-µm-thick sections. For immunostainings, samples were deparaffinized in xylene and rehydrated in a graded ethanol series. Antigen retrieval was performed in 0.1 M citrate buffer (pH 6.0) at 95°C. PMNs were stained with rat anti-mouse Ly6B.2 monoclonal antibody, clone 7/4 (AbD Serotec, UK). This was followed by staining with biotinylated donkey anti-rat IgG antibody (Agrisera, Sweden) and Alexa Fluor 647-conjugated streptavidin antibody (Thermo Fisher Scientific). Counterstaining was performed with DAPI (4′,6′-diamidino-2-phenylindole) (Thermo Fisher Scientific), and images were captured with a Zeiss Axio imager Z1. Images captured for quantification were taken with 10× magnification as tiles to cover the full sample. The tiles were aligned according to the DAPI filter prior to analysis in ImageJ software version 1.51. Manual adjustment of a set threshold was applied to each sample. Assuming fluorescence intensity is directly proportional to PMN density, a comparative analysis of each sample was performed.

### Quantification of metabolic activity.

Strains used in this study are listed in Fig. S1. Fungal growth curves were performed for the respective strains using the CellTiter-Glo luminescent cell viability assay. This assay is luciferase based and uses ATP to oxidize luciferin, which generates luminescence. The luminescent signal is proportional to the amount of ATP present. Samples were analyzed in a luminometer (Tecan Infinite F200) at the respective time points.

Assessment of colony growth was performed by plating 100 μl of 1 × 10^2^ cells on three YPD plates grown under normoxic, hypoxic, or anoxic conditions for 2 days. Afterwards, the diameters from 10 colonies from each plate were measured.

Metabolic activity of PMNs was assessed using the CellTiter-Glo assay similarly as described above. Lactate release by PMNs was assessed in supernatants of infected or uninfected samples (1 × 10^6^ cells/well) at 6 h using LactGen 2 (Roche Diagnostics).

### PMN cytokine release.

The release of cytokines by PMNs was quantified in 6 h supernatants of C. albicans infection. PMNs (1 × 10^5^ cells/well) were infected in 24 wells with C. albicans (multiplicity of infection [MOI] of 1) for 6 h. IL-8 release was determined using human IL-8 magnetic Luminex performance assay (R&D Systems) and read using a multiplex array reader Bio-Plex 200 system (Bio-Rad Laboratories). The cytokine concentration was based on the standard curve supplied by the manufacturer.

### ROS production of PMNs.

ROS production of PMNs was quantified by use of oxidative stress dye CMH(2)CFDA (Thermo Fisher Scientific). Briefly, PMNs were stained with the fluorescent dye for 10 min at room temperature, and unbound dye was removed by centrifugation and resuspension in fresh medium. Infection was performed in 96-well opaque plates with a cell density of 1 × 10^5^ cells/well for both fungal and human cells. Fluorescence intensity was measured at 1 h and 3 h after stimulation. Background fluorescence from unstained PMNs was subtracted from total fluorescence values prior to data analysis.

### PMN phagocytosis.

PMNs (1 × 10^5^ cells/well) were seeded into 96-well plates and incubated with pHrodo Red S. aureus bioparticle conjugate for phagocytosis (Thermo Fisher Scientific) for 1 h and 2 h (0.6 mM per well). The fluorescence intensity of beads (excitation 560/emission 585 nm) was measured in a plate reader (Fluostar, Omega, BMG). Acidized beads (sodium phosphate [100 mM; pH 4]) and PMNs with the blocked cytoskeleton (12.5 µM cytochalasin D) served as controls.

Alternatively, PMNs (5 × 10^5^ cells/well) were seeded into 24-well plates and infected with C. albicans *ENO-1* strain (MOI of 1) for 1 h and 3 h. Cells were collected and placed in tubes, and PMNs were stained using a labeled antibody directed against human CD66b (anti-CD66b labeled with PE; Biolegend, eBioscience) for 15 min on ice. To distinguish attached cells from true phagocytized cells, 0.4% trypan blue was added to all samples (60). Phagocytosis was assessed by flow cytometry, and phagocytic events were determined as the number of the double positive population according to the anti-CD66b-PE/*ENO-1* histogram. Prior granulocyte population was determined from the acquisition of approximately 100,000 events with a flow rate of 66 events/s after gating population according to the forward scatter (FSC)/side scatter (SSC) histogram.

### PMN receptor staining.

PMNs were stained using a labeled antibody directed against human CD66b (anti-CD66b-APC; BD Bioscience) and a labeled antibody directed against Dectin-1 receptor (Dectin-1 monoclonal antibody [GE2] FITC; Thermo Fisher Scientific) before or 2 h after *in vitro* infection with C. albicans. The granulocytes were analyzed by acquisition of approximately 50,000 events. Dectin-1 receptor internalization was determined by the loss of FITC signal after 2 h of infection compared to the signal of the corresponding uninfected control.

### Fungal killing by PMNs.

Fungal cells were seeded in 96-well clear bottom plates with a cell density of 1 × 10^5^ cells/well. PMNs were added at the same cell density. After 1 h and 3 h, 1% Tween 20 (Sigma-Aldrich) was added to wells to lyse PMNs, followed by the addition of CellTiter-Glo luminescent cell viability assay. Fungal viability was presented as a percentage of the ATP level normalized to ATP levels from fungal growth controls.

To assess fungal killing by PMNs using serial dilution and plating, fungal cells and PMNs were seeded in 1 × 10^6^ cells/well in 96-well plates for 1 h and 3 h. PMNs were then lysed with 10% Triton X-100, and fungal cells were adequately diluted and spread on YPD agar plates. Colonies were counted after 24 h to 48 h of incubation at 30°C. Fungal viability was presented as a percentage of CFU normalized to the values for fungal growth controls.

### Quantification of NET formation by PMNs.

PMNs (1 × 10^5^ cells/well) were seeded into 24-well plates containing coverslips which were coated with 0.001% poly-L-lysine (Sigma-Aldrich). PMNs were infected with C. albicans (MOI of 1) for 4 h and 6 h *in vitro*. Cells were fixed using 2% paraformaldehyde (PFA) and stored at 4°C. Cells were subsequently permeabilized with 0.5% (vol/vol) Triton X-100, blocked with 3% (wt/vol) BSA in PBS for 30 min, and incubated with pairs of primary and secondary antibodies as follows: anti-myeloperoxidase antibody (R&D Systems)/Alexa Fluor 633 rabbit anti-goat IgG and anti-neutrophil elastase (Dako)/Alexa Fluor 488 goat anti-mouse as well as anti-C. albicans (ProSci)/Alexa Fluor 568 donkey anti-rabbit IgG. This was followed by DNA staining with DAPI and coverslip mounting. Imaging data were acquired using a Nikon A1R laser scanning confocal microscope. NET quantification was performed as previously described using DAPI immunostained image samples from 10 biological replicates (59). The analyzed images contained 100 ± 30 cells per picture, and for each infection condition, a total of at least 700 cells were analyzed.

### Biofilm formation of different C. albicans strains.

Biofilm formation in C. albicans was determined similarly to the previously described method (44) with minor modifications. Biofilms were grown in wells of 96-well opaque plates for 20 h at a density of 1 × 10^6^ cells/well. C. albicans biofilms were washed prior to addition of fresh media for 2 h. Before starting the experiments, planktonic cells were added to a new set of wells at a similar cell density. Finally, PMNs were added to a final concentration of 1 × 10^5^ cells/well. After 4 h or 6 h, free DNA was quantified using 2.5 μM Sytox Green (Thermo Fisher Scientific) to determine NET release. PMNs lysed with Triton X-100 (10%) were used as the positive control of total free DNA. The fluorescence intensity was measured in a fluorescence plate reader (Fluostar Omega; BMG). Background fluorescence for controls containing C. albicans only was subtracted prior to data analysis.

Biofilm quantification was performed using the crystal violet method (61). Absorbance (590 nm) was measured in a plate reader, and pictures were obtained using a Zeiss Axiovert 25 microscope. For SEM imaging, the samples were fixed with 2% PFA and washed in PBS. Subsequently, the samples were dehydrated in a series of ethanol gradients, critical point dried, and coated with iridium (2 nm). The sample morphology was examined by field emission SEM (Carl Zeiss Merlin) using a secondary electron detector at an accelerating voltage of 4 kV and probe current of 120 Pa.

### Analysis of fungal cell wall.

C. albicans cell wall components were quantified before and after 3 h of incubation with or without PMNs (MOI of 1 [10^6^ cells/well]). Samples were analyzed using BD FACSLSRII with postacquisition data analysis using FlowjoV5 software. Staining of β-glucan was performed using 5 ng/μl Fc-hDectin-1a (Invivogen)/goat F(ab′)2 anti-human IgG conjugated to Alexa Fluor 488 (Invitrogen), Mannan was stained using 0.1 µg/ml concanavalin A conjugated to Alexa Fluor 594 (Thermo Fisher Scientific), and chitin was stained using 0.05% Calcofluor white (CFW) (Sigma-Aldrich).

For lactate-induced β-glucan masking, C. albicans (1 × 10^7^ cells/ml) were grown for 7 h either in RPMI or in anoxic supernatants from uninfected PMNs (1 × 10^6^ cells/ml). After 7 h, the cells were fixed with 2% PFA and stained the next day for β-glucan as described above and then analyzed by flow cytometry.

### C. albicans infection of C. elegans under hypoxic conditions.

C. elegans Bristol-N2 was maintained at 20°C until adult stage on nematode growth medium (NGM) plates. To collect eggs, fertilized C. elegans adults were incubated with alkaline lysis buffer while being shaken for 20 min at room temperature and subsequently washed twice with M9 buffer. To arrest the larvae to L1 stage, the C. elegans eggs were transferred for hatching on new NGM plates and incubated at 20°C for 20 h without food. L1 synchronized worms were transferred to fresh NGM with nonpathogenic E. coli OP50 for 48 h to mature up to the L4 stage. Five worms per well were then distributed on 24-well plates with NGM agar with 40 μM 5-fluoro-2′-deoxyuridine (FUDR) (Sigma) to prevent progeny production (55). Additionally, 500 μl HBSS medium (Lonza) was added to each well. Sets of five wells either contained 2 × 10^8^ E. coli OP50 cells/well or 3 × 10^5^ C. albicans SC5314 cells/well. Plates were incubated at 25°C in normoxic and hypoxic (1% O_2_) cell incubators with 5% CO_2_ for 48 h, and C. elegans survival was subsequently recorded with a Nikon SMZ1500 stereomicroscope.

### Antifungal disc diffusion assay.

C. albicans susceptibility to antifungal drugs was determined under normoxic and anoxic conditions using a disc diffusion assay. *Candida* cells grew for 20 h at 30°C, and five colonies were picked. Cells were counted and adjusted to a density to 5 × 10^6^ cells/ml. To obtain a confluent lawn of growth, 400 µl of this suspension was added to and spread on a YPD plate. Antibiotic disks containing amphotericin B (20 µg), fluconazole (25 µg), terbinafine (20 µg), and PBS were prepared and placed on the YPD agar. Plates were incubated for 20 h at 37°C, and the inhibition zone was measured.

### Statistical analysis.

All data are shown as mean ± SD. One-way analysis of variance (ANOVA) with Bonferroni’s posttest correction was applied when multiple groups were compared, and two-tailed Student’s *t* test was used for the analysis of two groups. For nonparametrically distributed data, the two-tailed Mann-Whitney U-test was used. Bars represent 95% CI. The statistical calculations were performed using GraphPad Prism Software 6.0 (GraphPad Software La Jolla, CA, USA), and for all analyses, *P* values of <0.05 were considered statistically significant.
