# Supplementary material for: Evasion of Immune Surveillance in Low Oxygen Environments Enhances Candida albicans Virulence
Source: mBio. 2018 Nov 6;9(6):e02120-18. doi: 10.1128/mBio.02120-18 (PMC6222133; doi:10.1128/mBio.02120-18)
Supplement: FIG S1 [file mbo005184149sf1.pdf]

**Fungal strains**	**Description**	**Genotype**	**Reference**
SC5314	*Candida albicans* standard wild-type strain		Gillum et al., 1984
Δ/Δ *efg1*	*C. albicans* yeast-locked mutant strain	*Δura3::imm434/Δura3::imm434 Δefg1::hisG/Δefg1::hisG-URA3-hisG*	Ramage et al.2002 / Sohn et al. 2003
Δ/Δ *efg1/EFG1*	*C. albicans* yeast-locked revertant strain	*Δura3::imm434/Δura3::imm434 Δefg1::hisG/Δefg1::hisG EFG1 URA3*	Ramage et al.2002
ENO1	*C. albicans* (CAI4) *ENO1* promoter GFP fusion.	CAI4 pENO1-GFP-CyC1t	Lopes et al., 2015
DSM 4238	*Candida tropicalis* standard wild-type strain		
**Clinical strains**			
Candida lusitaniae (46)	Blood isolate 2006	University Hospital of Umeå (NUS)	
Candida guilliermondii (47)	Blood isolate 2007	University Hospital of Umeå (NUS)	
Candida albicans (53)	Blood isolate 2007	University Hospital of Umeå (NUS)	
Candida glabratta (58)	Blood isolate 2007	University Hospital of Umeå (NUS)	
Candida krusei (25)	Blood isolate 2005	University Hospital of Umeå (NUS)	
Candida tropicallis (33)	Blood isolate 2006	University Hospital of Umeå (NUS)	
Candida tropicallis (56)	Blood isolate 2007	University Hospital of Umeå (NUS)	
Candida dubliniensis (80)	Blood isolate 2013	University Hospital of Umeå (NUS)	
