# Supplementary figures and images for: Evasion of Immune Surveillance in Low Oxygen Environments Enhances Candida albicans Virulence
Source: mBio. 2018 Nov 6;9(6):e02120-18. doi: 10.1128/mBio.02120-18 (PMC6222133; doi:10.1128/mBio.02120-18)

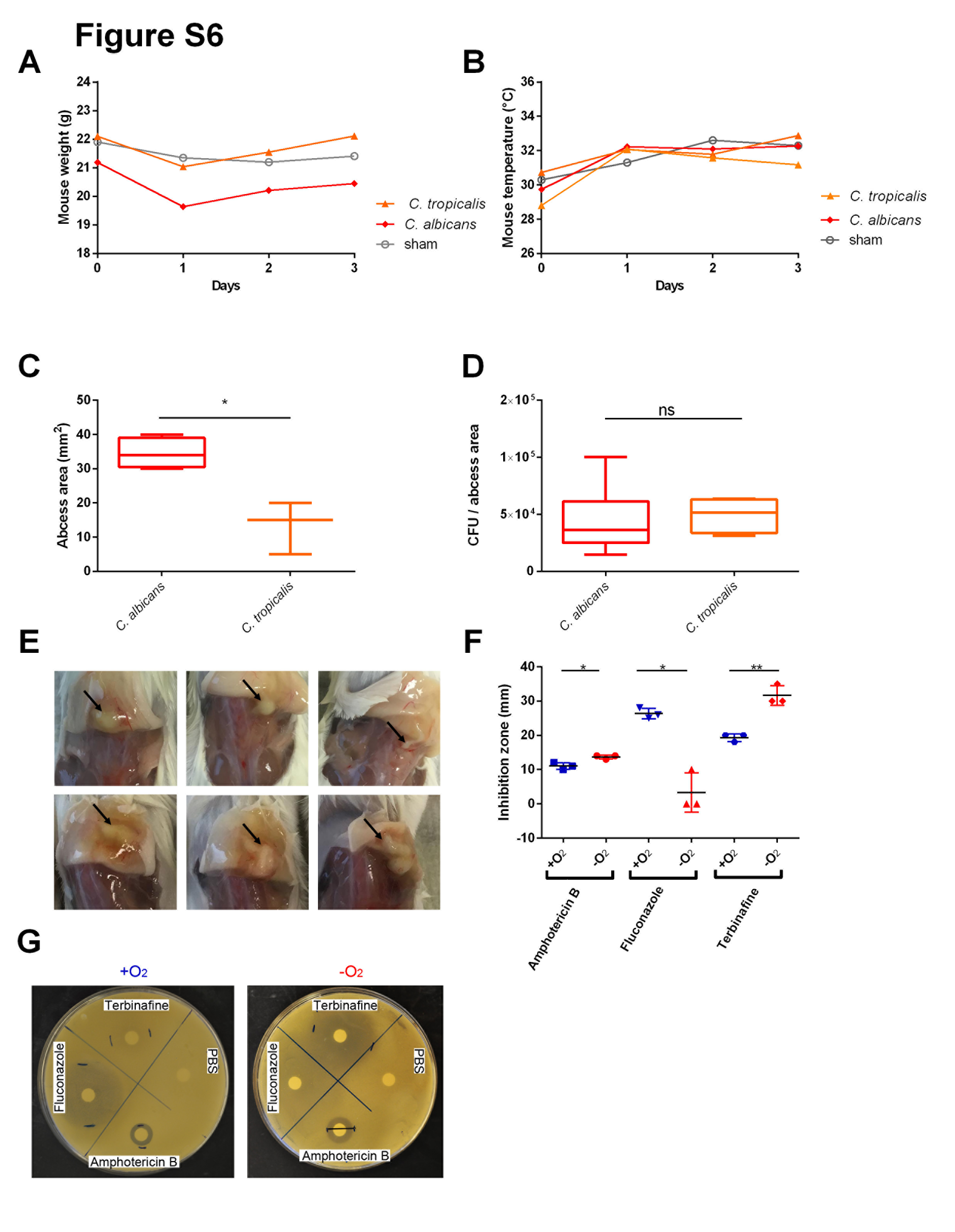

Supplement: FIG S6 [file mbo005184149sf6.tif]
